# Marketing Netcoatings for Aquaculture

**DOI:** 10.3390/ijms151018742

**Published:** 2014-10-17

**Authors:** Robert J. Martin

**Affiliations:** 318 Westover Drive, Asheville, NC 28801, USA; E-Mail: RJM948@aol.com

**Keywords:** aquaculture developments, aquaculture net coatings, fouling management, marketing, biocide registration, business development

## Abstract

Unsustainable harvesting of natural fish stocks is driving an ever growing marine aquaculture industry. Part of the aquaculture support industry is net suppliers who provide producers with nets used in confining fish while they are grown to market size. Biofouling must be addressed in marine environments to ensure maximum product growth by maintaining water flow and waste removal through the nets. Biofouling is managed with copper and organic biocide based net coatings. The aquaculture industry provides a case study for business issues related to entry of improved fouling management technology into the marketplace. Several major hurdles hinder entry of improved novel technologies into the market. The first hurdle is due to the structure of business relationships. Net suppliers can actually cut their business profits dramatically by introducing improved technologies. A second major hurdle is financial costs of registration and demonstration of efficacy and quality product with a new technology. Costs of registration are prohibitive if only the net coatings market is involved. Demonstration of quality product requires collaboration and a team approach between formulators, net suppliers and farmers. An alternative solution is a vertically integrated business model in which the support business and product production business are part of the same company.

## 1. Global Aquaculture

As the demand throughout the world increases for seafood, there is unsustainable pressure on wild fish stocks throughout the world. The world is rapidly approaching the limit that can be harvested from our oceans and in many cases fish populations such as tuna and cod [1] are in rapid decline due to overfishing. Only by severely limiting the amount of fish harvested each year can we hope to restore these species to levels where they can give us the sustainable harvest each year. In fact some populations such as bluefin tuna are already so depleted [2] that many feel only a complete shutdown of the fishery will result in the restoration of a sustainable population. As a result, the pressure on natural stock commercial fish farming has exploded to fill the gap between production and demand [3]. With the growth of aquaculture in the ocean environment, new problems arise that impact both the producers of fish and their suppliers in the fish farming market.

A large percentage of aquaculture production takes place in fresh water rivers and lakes where conditions can be closely controlled but is limited to species that can be raised in fresh water. The increasing popularity and declining stocks of marine fish has resulted in an increasing percentage of fish that are raised in bays, fjords, coves and open water environments creating new conditions for the industry to face. These include protection from predators, adequate water circulation to ensure healthy fish, pollution of the surrounding waters and sediment beneath the net, and most importantly maintaining high quality fish flesh. Over time, an industry has developed to support the fish farmers that will ensure healthy fish. These companies supply the fish containment structures including maintenance and repair, specialty feeds, net coating materials and certification programs for the harvested fish.

Over the past 20 years, the dramatic growth in farm raised fish to satisfy the market demand growth has resulted in larger containment pens for the fish. This has required improvement in all aspects including improved performance of the fish net coatings used to prevent fouling of the nets. While fouling conditions vary around the world, uncoated nets quickly become fouled with biological growth [4]. This fouling retards the flow of water through the net, and this can impact the growth rate and health of the fish. In Australia, some fish farmers who use an uncoated net are forced to change the nets every 7–10 days due to fouling. Around the world, most fish farmers are using some sort of net coating to extend the time between net changes. Ideally, a net would not have to be changed until the fish are ready to be moved to a larger net or harvested. With such an obvious need in the aquaculture market, it would seem that nets are an area that would attract innovation and new net coatings. However, there are numerous factors that make improved net coatings very difficult to introduce to the market. These factors are similar for introduction to the market of many fouling management products.

## 2. Marketing and Certification

Marketing into the industry is fairly complex as improved products sometimes directly impact companies in the supply chain. Consider the example of the company supplying nets coated with a cuprous oxide based antifouling coating to a fish farmer. Typically these coatings give good protection for 3–4 months after which the net must be changed to insure adequate water flow through the net. As the coating loses effectiveness, the fish farmer replaces the existing net with a net that has been newly coated. He returns the used net to the net supply company. The supply company cleans the net, makes necessary repairs and then recoats the net with a fresh antifouling coating.

The coatings are usually made by a coating formulation company, not the company that sells repairs and maintains the nets. If the coating formulation company comes up with an improved product that will last the fish farmer 6–7 months instead of 3 months, they have to convince the net coater to offer the improved product to the fish farmer. This can be a barrier to new products because if the net now lasts twice as long, it will cut the net supply company’s business in half. Therefore there is little incentive for them to promote an improved product. If the coating supplier tries to go directly to the fish farmer, it is possible it will cost the supplier his existing business to the net company. So to market an improved coating, the supply company must find a way to educate the fish farmers without ruining their business relationship with the net supplier. Education can result in pressure from the fish farmers that might induce the net coater to carry the product.

The situation is further complicated because the improved net formulation will usually contain new biocides which in some countries require registration. Agencies that require registration include the EPA in the US and the European Community [5] through the Biocide Product Directive in Europe. Convincing a biocide supplier for an existing biocide to spend the money to support registration is difficult because of the relatively small size of the aquaculture market and the cost due to the amount of data required to support registration. In fact, these cost barriers are so high it is almost impossible to get any company interested in developing new, more effective biocides for just the aquaculture market.

Another impact in the aquaculture market is the growth of certification societies involved with aquaculture who will certify that production meets a minimum standard of sustainability. This standard can include the use of biocides in net coatings. A good example is the standard developed by the World Wildlife Fund (WWF) for farm raised salmon through the Salmon Aquaculture Dialogue [6]. The standard took nearly a decade of meetings to develop. It involved producers and supply chain representatives, government officials, seafood buyers, non-governmental organizations (NGOs) and independent researchers.

Once the WWF sustainability standard was completed it was turned over to the Aquaculture Stewardship Council for implementation on a global basis. If a fish farmer wants to sell his product with this certification, he must now insure his products will meet this standard. While this standard impacts all aspects of farm raised salmon, let us examine how it impacts a supplier of antifouling biocides. This new standard means a net coater who wants to use a coating formulation containing antifouling biocides must ensure that the biocides are registered in either the US, Europe or Australia. This registration requirement ensures that there are adequate data to demonstrate the product is safe and does not persist in the environment or bio-accumulate in the fish. This requirement means the biocide supplier with a new biocide for this market will have to generate large amounts of environmental fate and toxicology data to obtain registration. This effort can cost the supplier millions of dollars. Since this is almost impossible to justify on the economics of just the fish net market alone, most biocides that are considered for use in the aquaculture market are ones used in ship antifouling coatings where the market is large enough to justify the cost of generating the required data for registration.

Once the biocide supplier has the data, it is necessary to demonstrate to regulators and fish farmers that the product does not bio-accumulate in fish or persist in the environment in the actual use of the coating on the fish nets. This means a co-operative effort is required between the biocide supplier, the coating company, the net supply company and the fish farmer. This adds more time and cost to the approval process. In the EU all coating formulations must be registered, further adding to the cost and complexity of introducing new products. It is estimated that it could cost well in excess of $100,000 to register a new coating formulation. This barrier to entry is a real deterrent to developing new antifouling technology that could help reduce the cost of farm raised fish by extending the time between net changes.

Often, the complex issues that are involved in selling an improved net coating deter companies from even trying to develop a new product for the market. Companies that have tried to develop new antifouling net coatings soon learn that this effort will cost a lot of money and take a significant amount of time to commercialize the new product. In many cases it is impossible to justify the cost to management.

## 3. Regulatory Requirements

Although this paper is discussing the aquaculture market, the barriers to entry of any new biocide in the marine market are significant because of regulatory requirements. While you could develop a product that would only be sold in countries where registration of biocides is not required, new regulations in Europe require treated articles entering Europe to only use biocides that have been registered under the Biocide Product Directive. This means a ship painted in China or Korea with an antifouling coating, any biocides used in the antifouling paint must be registered for use in the EU. The cost of generating all the toxicology and environmental fate data can range from $4M to $8M and will take a minimum of three years for data generation, with the registration process taking at least another 2–3 years. These regulatory barriers are important for researchers developing new biocidal products for the marine market to understand when beginning their efforts. It is also one of the reasons so much effort is being made to try and find non- or reduced biocidal solutions.

## 4. Solutions to the Conundrum

There is no easy way to enter the fouling management market with either an old or a new product. Therefore the best way to insure success is to have partners that are committed to supporting the development effort through to commercialization. For example if a biocide supplier has a new product that has potential to work well in the net market, they then need to find a coating formulator willing to help develop an improved antifouling coating. In addition this group will need a net company that supports the effort for improved performance and is willing to coat the nets needed for testing. Having fish farmers who will run the evaluation and allow monitoring of the trial, including fish collection and testing is also very important as is an agreement up front with the regional regulatory authorities on the testing program that will provide the data for registration of a biocide for aquaculture applications. It would also help to bring in an environmental group and reach agreement with them on the minimum data that would satisfy their concerns. If you can put together this group and get everyone involved willing to commit the time and money up front to get the product into commercial use, there is a good chance of success. While not an exact example, such a coalition helped bring about the elimination of organotins used as biocides when Arch Chemicals, International Paint, the World Wildlife Fund, Rohm and Haas, and the US EPA worked closely together on “The International Convention on the Control of Harmful Anti-Fouling Systems on Ships” [7]. A key step in the process was reaching agreement on what conditions in the treaty would satisfy industry, regulators, and environmentalists on the continuing use of biocides in antifouling paint market. Using this kind of coalition helped insure the success of the antifouling treaty and would be an excellent way to bring about innovation in the aquaculture market.

While such a coordinated team is a possible roadmap to success in aquaculture, another possible solution is the development of a vertically integrated company. This is a business model in which the support companies and fish farmers are in reality an integrated company whose profits depend upon efficiency and quality of production. While this would be difficult to accomplish as the size of aquaculture producers increase, there will be increasing incentive to back integrate if suppliers are unwilling to provide the most effective net coatings.
